# Characterisation of Dandelion Polyphenols and Their In Vitro Neuroprotective Effects During Simulated Digestion

**DOI:** 10.3390/foods15071126

**Published:** 2026-03-25

**Authors:** Chongting Guo, Bingchan Qu, Hongye Li, Xinru Li, Bowen Liu, Xingkui Wang, Youlin Xue, Chong Ning, Shan Wang, Jiasu Wu, Chang Tan

**Affiliations:** College of Light Industry, Liaoning University, Shenyang 110036, China

**Keywords:** dandelion, in vitro digestion, polyphenols, UPLC-QTOF-ESI-MS/MS, neuroprotective effect

## Abstract

Dandelion, a herb with medicinal and nutritional properties, was studied for the stability and neuroprotective effects of polyphenols from its flowers, roots, and leaves during in vitro simulated digestion. Using UPLC-QTOF-ESI-MS/MS, 84 phenolic metabolites were identified, with flavonoids being most abundant in flowers and phenolic acids in leaves and roots. In vitro neuroprotection assays revealed that leaf polyphenols exhibited the highest inhibition rates against acetylcholinesterase and lipoxygenase, while flower polyphenols showed the strongest scavenging activity against reactive nitrogen species. After simulated digestion, total phenol and flavonoid contents increased significantly. Notably, polyphenols from all dandelion parts demonstrated the strongest inhibition of acetylcholinesterase during the oral phase, while the small intestine phase showed the greatest inhibition of lipoxygenase and reactive nitrogen species. Moreover, leaf polyphenols maintained the highest inhibitory effect on acetylcholinesterase throughout all digestive stages, suggesting that dandelion leaves are a promising functional food for preventing neurodegenerative diseases.

## 1. Introduction

Neurodegenerative diseases, such as Alzheimer’s (AD) and Parkinson’s diseases, pose a global burden owing to ageing populations and current treatment limitations [1]. The Cholinergic Hypothesis, a classic pathogenic mechanism for AD, posits that the progressive loss of cholinergic neurons and the consequent decline in brain acetylcholine (ACh) levels are the core causes of cognitive impairment and memory loss in AD patients. Acetylcholinesterase (AChE) is the key hydrolase that catalyses the degradation of synaptic ACh; its abnormal overactivation accelerates ACh depletion, disrupting neural signal transmission and exacerbating AD pathological progression [2]. Key targets for these neurodegenerative diseases include acetylcholinesterase (AChE), lipoxygenase (LOX), and reactive nitrogen species (RNS). In addition, naturally occurring bioactive compounds, particularly polyphenols, have attracted attention for their potential neuroprotective effects. As a widely distributed wild plant, the dandelion (*Taraxacum officinale*) is renowned for its applications in traditional medicine. Dandelion contains numerous active compounds, including flavonoids, phenolic acids, and their derivatives. Polyphenols are important secondary metabolites that play crucial roles in these compounds. Previous studies have demonstrated that the neuroprotective properties of polyphenols may be used to effectively treat neurogenic diseases [3]. Recent studies indicate that elevated AChE activity decreases acetylcholine levels in the brain, disrupting neural signal transmission and potentially leading to cognitive disorders such as AD [4]. Neuronal processes associated with LOX are also a major cause of neurodegeneration, and abnormal fatty acid oxidase activity in the nervous system is linked to the occurrence and development of various neurodegenerative diseases. The inhibition of LOX activity by polyphenols may help mitigate neuroinflammation [5]. The accumulation of RNS during oxidative stress is also a potential cause of neurodegeneration, and elevated RNS levels can lead to neuronal death [6]. Among the compounds with neuroprotective activity, phenolic compounds are the most active [7].

In vitro digestive simulation is a sophisticated technique that simulates the physiological conditions of the human digestive tract to study the bioaccessibility, stability, and transformation of neuroprotective compounds. During simulated digestion in vitro, the total phenol and flavone contents, as well as the inhibitory activity of neuroprotection-related enzymes, undergo changes. These changes are crucial for understanding the bioactive transformations and neuroprotective potential of these substances. Murugan et al. [8] found that after simulated digestion in vitro, *Phoenix loureirii* fruit polyphenol compounds retained their inhibitory activity against AChE, suggesting their potential for treating gut-related diseases. Cavia et al. [9] found that in vitro digestion, regardless of the amylase used, resulted in the loss of phenolic compounds. Gutierrez-Grijalva et al. [10] reported that polyphenols are typically reduced during gastrointestinal digestion, indicating a lower bioaccessibility. However, during specific gastrointestinal digestion stages, the content of certain polyphenol compounds increases. Wu et al. [11] demonstrated that after simulated digestion of barley red koji tea soup in vitro, its comprehensive antioxidant index, *α*-glucosidase inhibitory activity, and pancreatic lipase inhibitory activity were significantly improved, and it had potential hypoglycaemic and hypolipidaemic effects.

This study aimed to investigate the polyphenol content, individual phenolic composition, and neuroprotective effects of different parts of the dandelion, thereby establishing a theoretical foundation for the development and application of dandelion polyphenols for neuroprotection.

## 2. Materials and Methods

### 2.1. Materials and Chemicals

The dandelions originated from Yuncheng, Shanxi, China, and were grown in sunny, fertile, and moist sandy loam soil, harvested in March 2023 and air-dried in the shade. Chromatographic-grade methanol and acetic acid (≥99%) were purchased from Thermo Fisher Scientific (Waltham, MA, USA). Polyphenol standards including protocatechuic acid, chlorogenic acid, caffeic acid, chicory acid, rutin, quercetin, luteolin, gallic acid, and rutin of 95–99% purity were purchased from Sigma-Aldrich (St. Louis, MO, USA). Folin–Ciocalteu reagent, ethanol, sodium carbonate (Na_2_CO_3_), sodium bicarbonate (NaHCO_3_), sodium nitrite (NaNO_2_), aluminium chloride (AlCl_3_), sodium hydroxide (NaOH), sodium dihydrogen phosphate (NaH_2_PO_4_), disodium hydrogen phosphate (Na_2_HPO_4_), ascorbic acid, ethyl acetate, thiocholine iodide, 2-nitrobenzoic acid (DTNB), AChE, LOX, huperzine A, linoleic acid, Tween-20, nitroprusside, *p*-aminobenzenesulfonic acid, naphthylethylenediamine dihydrochloride, and sulfanilamide were purchased from Sinopharm Chemical Reagent Co., Ltd. (Shanghai, China). Ultrapure water prepared using a Milli-Q system (Millipore, Bedford, MA, USA) was used throughout the experiments.

### 2.2. Sample Preparation

Dandelion powder was prepared by grinding the dried dandelion flowers, roots, and leaves, followed by filtration through a 60-mesh standard sieve. The extraction of polyphenols from dandelion flowers, roots, and leaves was performed, with slight modifications, as previously published [12]. Briefly, a 1 g portion of each dandelion sample was added to 40 mL of 60% ethanol in water (*v*/*v*) and extracted using ultrasound (40 kHz) for 60 min at 25 °C. The extracts were then centrifuged at 8000× *g* at 4 °C for 10 min. The supernatant was used as dandelion extract.

### 2.3. Total Polyphenol and Flavonoid Content

#### 2.3.1. Total Polyphenol Content (TPC)

The TPC of dandelion extracts was analysed by the method of Correa et al. [13], and some modifications were made to this method. To conduct the analysis, 1 mL of the dandelion extract or gallic acid solution was added to a 25 mL colorimetric tube and mixed with 1.0 mL of Folin–Ciocalteu reagent (1 mol/L). After allowing the mixture to stand for 5 min, 2.0 mL of Na_2_CO_3_ (12%) solution and water were added. The tube was then incubated in a water bath at 30 °C for a period of 90 min, and the absorbance of the solution was measured at 760 nm using a UV spectrophotometer (UV-2550 Shimadzu Corporation, Kyoto, Japan) at 760 nm. For the purpose of constructing calibration curves, gallic acid solutions with varying concentrations (2, 3, 4, 5, and 6 mg/mL) were prepared. (R^2^ = 0.9990). The TPC results were expressed in terms of milligrams of gallic acid equivalent (GAE) per 100 g of the dry extract (mg GAE/100 g). Each experiment was conducted in triplicate.

#### 2.3.2. Total Flavonoid Content (TFC)

The TFC in dandelion samples was assessed following the protocol outlined by Zhan et al. [14]. Briefly, 1 mL of dandelion extract or rutin standard solution was combined with 4.0 mL of 60% ethanol solution. Subsequently, 0.3 mL of a 5% NaNO_2_ solution was introduced, and the mixture was left to incubate for 8 min. Following this, 0.3 mL of 10% AlCl_3_ solution was added and incubated for 10 min. Finally, 4 mL of 4% NaOH was incorporated, and the volume was adjusted to 10 mL using a 60% ethanol solution. The tube was vortexed and allowed to stand undisturbed for 10 min, and the absorbance was measured at 510 nm. To generate a calibration curve, standard rutin solutions with concentrations of 2, 3, 4, 5, 6, and 7 mg/mL were prepared (R^2^ = 0.9992). The TFC results were reported as milligrams of rutin equivalent (RE) per 100 g of the dry extract (mg RE/100 g). The experiments were repeated thrice.

### 2.4. Individual Polyphenol Determination

Qualitative and quantitative analyses of dandelion polyphenols were conducted using an Agilent-7980A system (Agilent Technologies Inc., Santa Clara, CA, USA). The methodology employed was adapted from the protocol described by Guo et al. [15] with some modifications. The dandelion supernatants were mixed and subjected to filtration through a 0.45 µm mesh. The chromatographic column was a C18 (250 mm × 4.6 mm, 5 µm). The mobile solutions were methanol (A) and Milli-Q water with 2% acetic acid (B), and the gradient programme was 95:5 V(A)/V(B) at 0 min, 75:25 V(A)/V(B) at 20.0 min, 60:40 V(A)/V(B) at 35.0 min, 5:95 V(A)/V(B) at 45 min, and 95:5 V(A)/V(B) at 50.0 min. Analysis was performed at a flow rate of 1.0 mL/min, with the column temperature maintained at 40 °C. The detection wavelength was 325 nm, and the injection volume of each sample was 10 μL. The quantification of individual polyphenols was achieved through the use of calibration curves and external standards. The findings are presented as the mean value ± standard deviation, expressed in milligrams per 100 g of the sample (mg/100 g).

### 2.5. Metabolomic Analysis

Metabolomic analysis of dandelion flowers, roots, and leaves was performed on an Agilent-G6545 liquid chromatography with quadrupole-time-of-flight mass spectrometry/mass spectrometry (LC-Q-TOF-MS/MS) system (Agilent Technologies Inc., CA, USA) coupled to an electrospray ionisation (ESI) source, in accordance with our previous research [16]. The column was an ACQUITY UPLC (ultra-performance LC) BEH C18 column (2.1 mm × 100 mm, 1.7 μm; Waters Corp., Milford, CT, USA) set at 30 °C with a C18 guard. A sample injection volume of 5 μL was used, and the flow rate was set at 0.4 mL/min. The mobile phases used consisted of A (0.1% formic acid in Milli-Q water, *v*/*v*) and B (0.1% formic acid in acetonitrile, *v*/*v*), and the gradient mixture of solvents was optimised as follows: 7–30% B for 0–4.5 min, 30–100% B for 4.5–6.5 min, 100% B for 6.5–7.5 min, 100–7% B for 7.5–8 min, and 7% B for 8–10 min. The column was re-equilibrated between injections for 3 min using the initial mobile phase conditions. The MS instrument was operated in negative ionisation mode (ESI^−^), and the MS range (*m*/*z*) was set at 50–1700. The system autosampler was maintained at 10 °C. Dynamic background subtraction was used to characterise the information-dependent acquisition of the UPLC-QTOF-ESI-MS/MS analysis.

Data were collected using the Agilent Mass Hunter Qualitative Software (B.07.00) (Agilent Technologies, USA). The untargeted metabolomic data were transformed to the ABF format using the Analysis Base File converter software (v1.2, Agilent Technologies). Data processing, including deconvolution, peak picking, alignment, and identification, was performed using the MS-DIAL software (v4.9.221218). Unknown metabolites, duplicated metabolites and isotopes, metabolites with a peak height less than three times that observed in blank samples, and metabolites with a peak height below 1000 units were excluded from the final metabolite list.

### 2.6. Neuroprotection Analysis

#### 2.6.1. AChE Inhibitory Activity

The AChE inhibitory activity was evaluated following a modified version of a previously established protocol [17]. Briefly, 25 µL of dandelion extract, prepared at various concentrations ranging from 0.2 to 1.0 mg/mL, was added to each well of a 96-well plate. Subsequently, 50 µL of PBS buffer (0.1 mol/L, pH 8) and 25 µL of 0.3 U/mL enzyme (AChE in buffer) were introduced into each well. After a 20 min incubation at 4 °C, 125 µL of 0.6 mg/mL DTNB (5,5-dithio-bis-(2-nitrobenzoic acid)) in buffer and 25 µL of acetylthiocholine iodide were added to the mixture and the reaction was conducted at 37 °C for 20 min. Huperzine A was used as the reference inhibitor. The absorbance of the mixture was measured at 405 nm using a microplate reader. Results were calculated using the following equation:

$$AChE\;inhibition\;rate\;(\%)=\left(1-\frac{A_{3}-A_{4}}{A_{1}-A_{2}}\right)\times 100\%$$

where A_1_ is the absorbance of the blank measurement group (where no sample was added), A_2_ is the absorbance of the blank control group (without sample or AChE), A_3_ is the absorbance of the sample group, and A_4_ is the absorbance of the sample control group (without adding AChE in the sample test group).

#### 2.6.2. LOX Inhibitory Activity

LOX inhibitory activity was determined following a protocol adapted from Paun et al. [18] with minor adjustments. Briefly, 1 µL of dandelion extract at different concentrations (0.2–1.0 mg/mL) and 20 µL of LOX in buffer were placed in a 96-well plate, and the mixture was incubated in a 30 °C water bath for 30 min. Afterwards, 150 µL of substrate (Tween-20 at a concentration of 0.5 mL/L in a 0.2 mol/L boric acid buffer) was added, followed by linoleic acid at a concentration of 0.54 mL/L. The solutions were thoroughly mixed, the pH of the buffer was adjusted to 9 with 1 mol/L sodium hydroxide, and it was incubated at 30 °C for 3 min. To halt the reaction, 500 µL of anhydrous ethanol was added, followed by 500 µL of distilled water, which was then mixed for determination. Ascorbic acid served as the reference inhibitor. The absorbance of the resulting mixture was measured at 234 nm using a microplate reader. The LOX inhibition rate was subsequently calculated using the provided equation:

$$LOX\;inhibition\;rate\;(\%)=(1-\frac{B_{1}-B_{2}}{B_{3}-B_{4}})\times 100\%$$

where B_1_ is the absorbance of the sample group, B_2_ is the absorbance without sample addition, B_3_ represents the absorbance measured in the sample control group (without adding LOX in the sample test group), and B_4_ is the absorbance without sample addition (first adding anhydrous ethanol and then adding the substrate).

#### 2.6.3. RNS Scavenging Activity

RNS scavenging activity was determined following the method outlined by Suluvoy et al. [19]. In summary, 1 mL of dandelion extract, prepared at various concentrations (0.2–1.0 mg/mL), was combined with 1 mL of 10 mM sodium nitroprusside solution (dissolved in 0.5 mM PBS, pH 7.4). The mixture was then incubated at room temperature (25 ± 1 °C) under light for 150 min. Subsequently, 1 mL of sulfanilic acid reagent (0.33% in 20% glacial acetic acid) was added, and the mixture was incubated for 10 min. Subsequently, 1 mL of naphthalene ethylenediamine hydrochloride solution (0.1% *w*/*v*) was added, and the reaction mixture was left to stand at room temperature for 30 min. The absorbance of the resulting solution was measured at 540 nm. Similar procedures were repeated using different concentrations of ascorbic acid. The RNS clearance rate was then calculated using the provided equation:

$$RNS\;clearance\;rate\;(\%)=(1-\frac{A_{2}-A_{1}}{A_{0}})\times 100\%$$

where A_1_ is the absorbance of the sample group, A_2_ is the absorbance of the blank control group (without the sample), and A_0_ is the absorbance of the sample control group (without the addition of naphthalene ethylenediamine hydrochloride solution in the sample test group).

### 2.7. In Vitro Simulated Digestion

The in vitro simulated oral, gastric, and intestinal digestion procedures were performed as previously described [17], with some modifications.

#### 2.7.1. Oral Digestion

The dandelion extracts (8 mg/mL, 25 mL) were mixed with 25 mL of oral digestive juice (NaCl 8.006 mg/mL, KCl 0.201 mg/mL, Na_2_HPO_4_·12H_2_O 3.581 mg/mL, KH_2_PO_4_ 0.204 mg/mL, and *α*-amylase dissolved to 220 U/mL), and the mixture was adjusted to pH 7 ± 0.02 with NaOH. The oral digestion procedure was simulated by oscillating the mixture at a rate of 100 rpm for 10 min at 37 °C.

#### 2.7.2. Gastric Digestion

The pH of the oral digestive sample was immediately adjusted to 2.0 ± 0.02 by using 1 mol/L HCl. The oral digestive sample (30 mL) was mixed with 30 mL of gastric simulated digestive juice (NaCl 3.10 mg/mL, KCl 1.10 mg/mL, CaCl_2_ 0.150 mg/mL, NaHCO_3_ 0.60 mg/mL, and 0.67 g pepsin) and then oscillated at a rate of 100 rpm for 2 h at 37 °C.

#### 2.7.3. Intestinal Digestion

The pH of the gastric digestive sample was adjusted to 7 ± 0.02 with Na_2_CO_3_. The gastric digestive sample (30 mL) was mixed with 30 mL of intestinal simulated digestive juice (NaCl 5.40 mg/mL, KCl 10.65 mg/mL, CaCl_2_ 0.33 mg/mL, 7 g pancreatin, 4% sodium cholate solution, 200 mL bile salt solution, and 13 mg trypsin) and oscillated at a rate of 100 rpm for 2 h at 37 °C.

#### 2.7.4. Sample Collection

Samples from each digestion stage were centrifuged at 10,000× *g* for 10 min at 4 °C, and the supernatants were collected for subsequent analyses.

### 2.8. Statistical Analysis

All the assays were conducted in triplicate, and the results are presented as means ± standard deviations. Statistical significance was determined by one-way analysis of variance (ANOVA) with post hoc Tukey’s test using IBM SPSS Statistics software (version 19, SPSS Inc., Chicago, IL, USA). A *p*-value threshold of less than 0.05 (*p* < 0.05) was used to determine statistical significance. Principal component analysis (PCA) and partial least squares discriminant analysis (PLS-DA) were performed and heat maps were generated using the MetaboAnalyst website (https://www.metaboanalyst.ca/home.xhtml).

## 3. Results and Discussion

### 3.1. TPC, TFC, and the Main Polyphenols in Dandelion Extracts from Different Plant Parts

The TPC, TFC, and main polyphenolic components of dandelion flowers (DFs), roots (DRs), and leaves (DLs) are presented in Table 1. The results indicated that the TPC in DL obtained through ultrasound-assisted ethanol extraction was the highest (3986.67 ± 413.70 mg GAE/100 g), followed by that of the DF (3354.00 ± 161.12 mg GAE/100 g), with the DR having the lowest content (1876.00 ± 341.29 mg GAE/100 g). This finding aligned with the outcomes reported by Haghighi et al. [20], who showed that the TPC differed among the leaves and flowers of *Vitex pseudo-negundo*, including dandelion (2960–3290 mg/100 g). The TFCs in the extracts of DF, DR, and DL exhibited similar trends to their TPCs, and were 2411.00 ± 249.94, 1512.00 ± 34.72, and 3250.00 ± 118.04 mg RE/100 g, respectively. This finding aligns with that of a previous report, which indicated that TPC and TFC in DL were higher than those in DF and DR [21].

The leaves and petals of dandelions contain diverse polyphenols, including cinnamic acid derivatives and flavonoids, which exhibit various biological activities [22]. The dominant polyphenols in the different parts of the dandelion, including protocatechuic acid, chlorogenic acid, caffeic acid, chicoric acid, rutin, luteolin, and kaempferol, were qualitatively and quantitatively analysed (Table 1). These polyphenols have been listed as key phytochemical ingredients of dandelion in a previous study [23]. The composition and content of these polyphenols showed notable differences among the DF, DR, and DL. The contents of protocatechuic acid, chicoric acid, rutin, caffeic acid and chlorogenic acid of dandelion in different parts ranged from 284.66 to 1899.88 mg/100 g, 35.66 to 116.43 mg/100 g, 3.00 to 17.00 mg/100 g, 0.99 to 2.04 mg/100 g, and 0.12 to 2.11 mg/100 g, respectively. Specifically, DL contained the highest levels of protocatechuic and chicoric acids, whereas the contents of rutin and caffeic acid were the highest in DF. Quercetin was not detected in DL, and luteolin was not detected in DR in this study.

### 3.2. Characterisation of Polyphenol-Related Metabolites from Dandelion

Table 2 summarises the chemical profiles of *T. officinale* extracts (DF, DR, and DL) obtained by UPLC-ESI-Q-TOF-MS analysis, including the identification confidence levels (ICLs) of each metabolite. The primary objective of this metabolomic analysis was to comprehensively characterise the polyphenolic profile of dandelion, thereby identifying potential neuroprotective compounds. To ensure the reliability and reproducibility of metabolite identification, ICLs were assigned based on the hierarchical system proposed by Schymanski et al. [24], which was optimised from the minimum reporting standards of the Metabolomics Standards Initiative (MSI) [25]. The ICL definitions are as follows: (1) Level 1: Confirmed structure via matching with commercial reference standards (consistent MS, MS/MS spectra, and retention time). (2) Level 2a: Probable structure with unambiguous spectral matching to public/commercial libraries. (3) Level 2b: Probable structure deduced from diagnostic MS/MS fragments, ionisation behaviour, or parent compound information (no standards/libraries available). (4) Level 3: Tentative candidate(s) with insufficient evidence for a single exact structure (e.g., positional isomers). Levels 4 (only molecular formula confirmed) and 5 (only exact *m*/*z* measured) were not involved in this study.

A total of 84 compounds were tentatively identified and classified into four distinct chemical subclasses. Among these, 38 phenolic acids (44.7%) and 39 flavonoids (45.9%) were the predominant components. The detailed spectral characteristics of key metabolites are as follows: Phenolic acids were dominated by caffeoylquinic acid derivatives (e.g., 1,3-dicaffeoylquinic acid, chlorogenic acid) and hydroxybenzoic acid derivatives (e.g., protocatechuic acid, gallic acid). Flavonoids included flavonoid aglycones (e.g., luteolin, quercetin) and glycosides (e.g., astragalin, quercetin 3-*O*-arabinoside). Four coumarins (e.g., 5,7-dihydroxycoumarin, isoimperatorin) were identified based on lactone ring cleavage fragments (e.g., *m*/*z* 135.02 for 5,7-dihydroxycoumarin), while pentagalloylglucose (the only tannin) was confirmed by its high molecular weight (*m*/*z* 769.09) and galloyl group fragmentation. This aligns with earlier findings that underscore the prominent presence of flavonoids in DF and DL [26]. Notably, our study identified a significantly higher number of polyphenolic compounds than the 2022 investigation that used UHPLC-ESI-MS/MS to examine Shandong dandelion extracts and detected only 17 major polyphenol classes [27]. This discrepancy is likely attributable to differences in analytical methodologies (e.g., enhanced resolution of UPLC-TOF-MS) or advancements in the comprehensiveness of databases.

During metabolomic data processing, metabolites with a peak height below 1000 units or less than three times that of blank samples were excluded (Section 2.5). For free phenolic acids (e.g., protocatechuic acid, caffeic acid) and flavonoids (e.g., rutin, quercetin) with high polarity or low abundance, their MS signals were easily suppressed by matrix components (e.g., high-abundance flavonoid glycosides and dicaffeoylquinic acid derivatives). Additionally, partial protocatechuic acid and quercetin in dandelion exist in bound forms (e.g., glycosides, esters), which were identified as derived metabolites in Table 2 (e.g., quercetin 3-*O*-arabinoside, quercetin 3-*O*-malonylglucoside) but not detected as free forms via UPLC-QTOF-MS/MS. However, these free phenolics were successfully quantified by HPLC-UV due to its high selectivity for targeted compounds and resistance to matrix interference.

Furthermore, eight polyphenols with significant antioxidant activity, including caffeic acid, chlorogenic acid, and luteolin, were consistently identified in all dandelion-tissue-derived extracts. This finding supports the existing literature on the bioactivity of dandelion [28].

### 3.3. Multivariate Analysis

**Figure 1:**
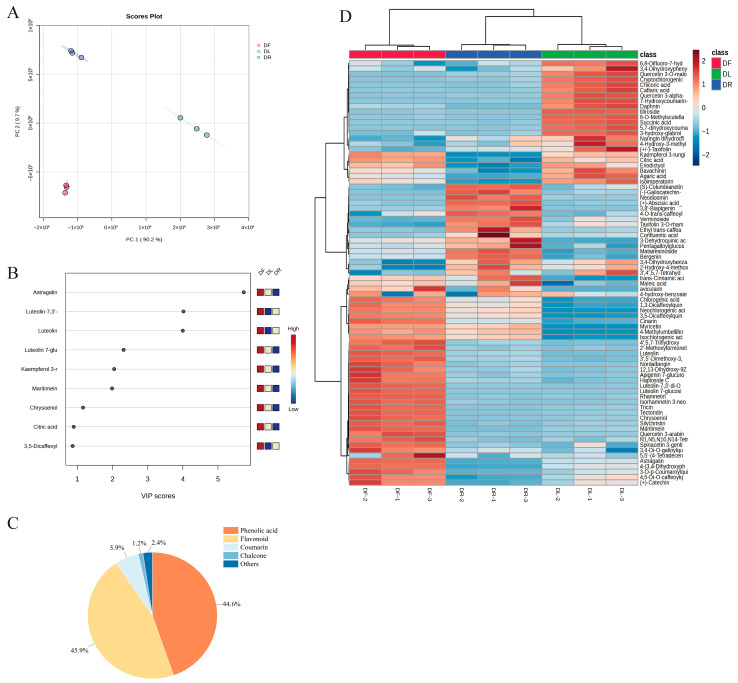
Multivariate statistical analysis showing the projection of metabolites from dandelion extracts: (**A**) principal component analysis (PCA); (**B**) partial least squares discriminant analysis (PLS-DA); (**C**) main classification pie chart of metabolites; (**D**) HeatMap.

The metabolomic profiling of tissue-specific extracts was performed using PCA, PLS-DA, and hierarchical clustering heat maps (Figure 1). PCA revealed two principal components (PC1 and PC2) that accounted for 90.2% and 9.7% of the total variance, respectively (Figure 1A). The DF samples clustered in the PC1-negative region and exhibited significant dispersion, indicating heterogeneous metabolite profiles. In contrast, the DF and DR extracts were located in the negative PC1 values, with the DF samples skewed toward negative PC2 values. Both DL and DR exhibited tight clustering, suggesting high analytical reproducibility, whereas DF dispersion indicated tissue-specific metabolic variability.

PLS-DA identified nine metabolites with variable importance in projection (VIP) scores exceeding one. Among these, seven metabolites—astragalin, luteolin-7, 3′-diglucoside, luteolin, kaempferol 3-rhamnoside, maritimein, chrysoeriol, and luteolin 7-glucoside—served as key discriminators (Figure 1B). Astragalin, which had the highest VIP score, exhibited significantly elevated concentrations in DF compared to those in DL and DR, as illustrated by the colorimetric gradient, where red denotes high concentrations, while blue signifies low concentrations. The compound class distribution confirmed that flavonoids (45.9%) and phenolic acids (44.6%) were the predominant constituents (Figure 1C).

Hierarchical clustering heat maps confirmed the accumulation of tissue-specific metabolites (Figure 1D). Three biological replicates per sample were analysed, with colour gradients ranging from blue to red, indicating metabolite abundance. Clustering distinctly segregated the samples into two groups: (1) flavonoid-rich DF (e.g., luteolin and quercetin) and (2) phenolic-acid-dominant DL/DR (e.g., caffeic acid and gallic acid). This alignment with PCA and the existing literature [29] underscores the organ-specific metabolic specialisation observed in dandelions.

### 3.4. In Vitro Neuroprotective Effects

#### 3.4.1. AChE Inhibitory Activity of Dandelion Polyphenols

**Figure 2:**
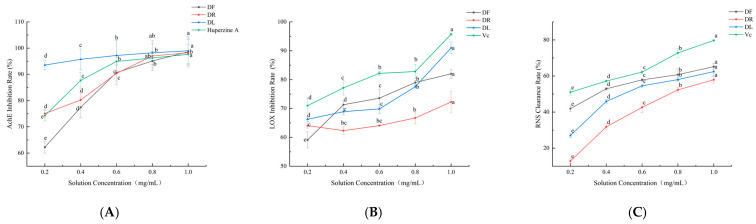
The inhibitory activity levels of dandelion extract: (**A**) acetylcholinesterase activity levels (AChE); (**B**) lipoxygenase activity levels (LOX); (**C**) RNS activity levels (RNS). Different letters indicate significant differences between groups (*p* < 0.05).

The neuroprotective potential of dandelion, particularly its antioxidant and anti-cholinesterase activities, has been increasingly recognised and summarised in recent reviews [30,31]. In line with these established properties, our study further dissects the AChE inhibitory effects of different dandelion parts. Although the data vary, several studies have shown that oxidative stress products have deleterious effects on specific cells in AD [32]. Plant-derived polyphenols (e.g., ellagitannin, ellagic acid, and urushiol) antagonise oxidative stress and reduce AChE activity, providing a natural alternative for AD treatment [33]. As shown in Figure 2A, dandelion extract exhibited concentration-dependent AChE inhibition, and the inhibition of polyphenols in DF, DR, and DL were progressively enhanced with increasing concentrations. When tested at 0.2 mg/mL, the inhibition rates of DF, DR, and DL were 62.24 ± 2.27%, 75.06 ± 1.61%, and 93.55 ± 1.78%, respectively, indicating that the concentration-dependent order of the inhibitory efficacy at this time was DL > DR > DF. By providing targeted data on neuroprotective polyphenols in a specific ecotype, consistent with Tanasa et al. [34], we confirmed that dandelion leaves are a rich source of phenolic acids. The inhibition rate of DL tended to stabilise above 0.6 mg/mL, whereas the stabilisation of DR started at 0.8 mg/mL. When tested at 1.0 mg/mL, the final inhibition of DF, DR, and DL reached 98.59 ± 4.66%, 98.22 ± 5.33%, and 98.98 ± 5.46%, respectively. Notably, at 1.0 mg/mL, the order of magnitude of inhibition shifted in a concentration-dependent manner, finally showing DL > DF > DR. This phenomenon may originate from a non-competitive inhibition mechanism where the inhibitor attaches to the enzyme, irrespective of whether the active site is occupied by the substrate, inducing a conformational change that reduces catalytic activity. This explains why plant parts with higher polyphenol content exhibited stronger AChE inhibition at high concentrations. This conclusion is supported by a study performed Murugan et al. on the fruit and flower stalk extracts of *Phoenix loureirii*, which showed that both gastric- and pancreatic-digested polyphenols inhibited AChE through a non-competitive inhibitory mechanism [8]. Critically, this study confirmed that the TPC was positively correlated with inhibitory potency, consistent with the observed order of potency of DL > DF > DR at 1.0 mg/mL. DL polyphenols efficiently inhibited AChE through non-competitive inhibition (93.55 ± 1.78 → 98.98 ± 5.46%), with TPC dominating the efficacy, providing a rationale for natural therapies for AD.

#### 3.4.2. LOX Inhibitory Activity of Dandelion Polyphenols

LOX catalyses the oxidation of polyunsaturated fatty acids to fatty acid hydroperoxides, leading to aberrant lipid metabolism and oxidative stress. Inhibition of LOX activity reduces the production of oxidised lipids [35]. As shown in Figure 2B, LOX scavenging by DF and DL was gradually enhanced with increasing polyphenol concentrations, which may be because the inhibitory effect of protocatechuic acid was enhanced with increasing concentration. The inhibitory activities of DL, DF, and DR polyphenolic compounds against LOX were 91.00 ± 2.07%, 82.01 ± 1.58% and 72.25 ± 3.59%, respectively, at the experimental concentration of 1 mg/mL. DL contained the highest TPC (3986 ± 413.70 mg/100 g) and TFC (3250 ± 118.04 mg/100 g) (Table 1) which may be due to its high LOX inhibition. Deuchande et al. showed that protocatechuic acid inhibited LOX in a concentration-dependent manner (IC50 0.38 mg/mL; complete inhibition at 1 mg/mL) [36]. This is consistent with the current study’s results. In contrast, the LOX clearance of DR extract exhibited an initial decline followed by an increase, probably due to the presence of the antagonistic effect of quercetin and chlorogenic acid. Similarly, Olszowy-Tomczyk et al. [37] investigated the antioxidant effect of a mixture of quercetin and chlorogenic acid and found that, using methanol as a solvent, the quercetin–chlorogenic acid mixture (50:50 *v*/*v*) exhibited an antagonistic effect with an F-value of 19.51, much higher than the critical value of 7.71. While previous reviews have highlighted the antioxidant components of dandelion [38], our study provides a quantitative comparison of LOX inhibition across different plant parts and identifies potential antagonistic effects in root extracts, adding a layer of complexity to the understanding of dandelion’s anti-inflammatory mechanism. Subsequent studies are needed to resolve polyphenol interactions and optimise formulations to enhance the potential for natural LOX inhibitor development.

#### 3.4.3. RNS Scavenging Activity of Dandelion Polyphenols

Nitric oxide (NO) regulates vasodilation and neural signalling at physiological concentrations; however, excess NO generates peroxynitrite (ONOO-) with superoxide anions, leading to oxidative damage associated with the development of chronic inflammation (e.g., arthritis and atherosclerosis) and malignancy (e.g., colorectal and lung cancers) [39]. The detection of RNS levels can reveal their molecular mechanisms in specific pathologies and inform the development of targeted antioxidant therapies. As illustrated in Figure 2C, there was a significant variation in RNS inhibition at a lower concentration (0.2 mg/mL) of DF, DL, and DR, where the inhibition rate was in the order of DF (41.92 ± 1.63%) > DL (27.03 ± 1.22%) > DR (12.84 ± 1.22%). At a higher concentration (1 mg/mL), the difference was reduced, with DF (65.22 ± 1.80%) > DL (62.44 ± 2.25%) > DR (57.93 ± 1.87%), and the difference in RNS inhibition of DF, DL, and DR decreased. Ribeiro et al. showed that RNS inhibition in cauliflower stem bark (0.95 ± 0.3 μg/mL) and flowers (1.8 ± 0.2 μg/mL) had significantly higher RNS inhibitory activity than the fruit (3.6 ± 0.2 μg/mL) [40]. The IC50 values of each site were close to saturation (flower: 4.9 ± 0.3; stem bark: 5.8 ± 0.4; fruit: 5.6 ± 0.2 μg/mL) at high concentration (HCO_3_^−^-containing), and the difference was reduced. This aligns with our study’s findings. Differences in phenolic composition dominate and inhibition rates may diverge at low concentrations, whereas synergistic effects are enhanced or active ingredients are saturated at high concentrations and differences are reduced [40]. DF has good potential for application as a targeted antioxidant drug at low concentrations.

### 3.5. Changes in TPC and TFC In Vitro Simulated Digestion

Table 3 presents the TPC and TFC in the flowers, roots, and leaves of dandelions during the simulated digestive stages in vitro, namely the oral, gastric, and small intestine phases. The results indicated that during the simulated digestion process, the TPC in each part of the dandelion followed the order of small intestine > oral > stomach. In the oral stage, the TPCs of DF, DR, and DL were 3902.00 mg/100 g, 2824.67 mg/100 g, and 4476.00 mg/100 g, respectively. Compared to their pre-digestion levels, the TPC of all dandelion parts increased slightly. This may be attributed to the interaction between polyphenols and *α*-amylases [41]. After simulated gastric digestion, the total polyphenol release was 2467.00 mg/100 g, 2072.00 mg/100 g, and 3796.00 mg/100 g for DF, DR, and DL, respectively. A decrease in TPC was observed in all parts because the pH of the stomach was low, the solubility of phenols significantly decreased, and some phenols were destroyed [42]. Notably, although gastric acidification does not directly induce the release of bound phenolics (which is the main reason for the TPC increase), the acidic conditions of the stomach play a critical indirect role: gastric acid disrupts the hydrogen bonds, glycosidic linkages and cellulose–pectin network of the dandelion plant cell wall matrix, leading to the loosening and partial disintegration of the cell wall structure. This structural damage pre-treats the plant matrix and creates a structural basis for the subsequent massive release of bound phenolics in the intestinal digestion phase. Following simulated intestinal digestion, the total polyphenol release was 7620.00 mg/100 g, 6726.00 mg/100 g, and 9398.00 mg/100 g for DF, DR, and DL, respectively. The increase in TPC across all dandelion parts was consistent with previous findings [43]. This remarkable TPC elevation is primarily attributed to the release of bound phenolics from the plant cell wall matrix: the intestinal digestive fluid contains a variety of hydrolases (lipases, proteases, amylases) and bile salts, which further decompose the pre-loosened cell wall structure, break the covalent and non-covalent bonds between phenolic compounds and macromolecules (polysaccharides, proteins, lignin) in the matrix, and thus release the large amount of bound phenolics embedded in the cell wall into the digestive fluid [44].

Across the simulated digestion stages in vitro, the TFC of each part of the dandelion was in the order of mouth > small intestine > stomach. After simulated oral digestion, the TFC in the DL was 3601.33 mg/100 g, which was slightly higher than that in the undigested state. In contrast, the release of total flavonoids in DF and DR was 1514.00 mg/100 g and 793.00 mg/100 g, respectively, showing a decrease compared to the undigested state. This decrease may be due to *α*-amylase causing some degree of flavonoid digestion in dandelions, which aligns with Zheng et al.’s results [45]. After simulated digestion in the stomach, the total flavonoid amounts released were 698.00 mg/100 g, 380.00 mg/100 g, and 2885.67 mg/100 g for DL, DF, and DR, respectively. The flavonoid content in all parts of the dandelion decreased, which is consistent with the conclusions drawn by Lucas-Gonzalez et al. [46]. After simulated digestion in the small intestine, the total flavonoids released were 1541.00 mg/100 g, 895.00 mg/100 g, and 3245.67 mg/100 g for DL, DF, and DR, respectively. The TFC in all parts of the dandelion increased slightly after digestion, possibly because trypsin and bile broke the ester bonds on the cell wall and facilitated the release of flavonoids [47].

In summary, after simulated digestion in vitro, the order of total phenols and flavonoids in each part of the dandelion plant was as follows: DL > DF > DR.

### 3.6. Changes in Phenolic Contents In Vitro Simulated Digestion

The main polyphenolic components in the DF, DR, and DL of dandelions during the simulated digestion stages (oral, stomach, and small intestine) in vitro are shown in Table 3.

Variations in the total phenols, total flavonoids, and related activities in the digested samples may be due to changes in specific phenolic substances [48]. Four phenolic acids and nine phenolic acid standards were identified by HPLC. During oral digestion, the most abundant compounds released were protocatechuic acid (133.48 mg/100 g) and chicoric acid (7.19 mg/100 g) in DF, and protocatechuic acid (43.13 mg/100 g) and chicoric acid (9.90 mg/100 g) in DR. In DL, the most abundant compounds were protocatechuic acid (932.00 mg/100 g) and chicoric acid (61.99 mg/100 g). During gastric digestion, the primary compound released was caffeic acid (0.15 mg/100 g and 0.10 mg/100 g, respectively) in DF and DR, while in DL, the primary compounds released included protocatechuic acid (350.48 mg/100 g) and caffeic acid (0.07 mg/100 g). In the intestinal digestion stage, the primary compound(s) released by DF was chicoric acid (0.38 mg/100 g), by DR was caffeic acid (0.06 mg/100 g), and by DL were protocatecholic acid and chicoric acid (95.58 mg/100 g and 6.63 mg/100 g, respectively).

**Figure 3:**
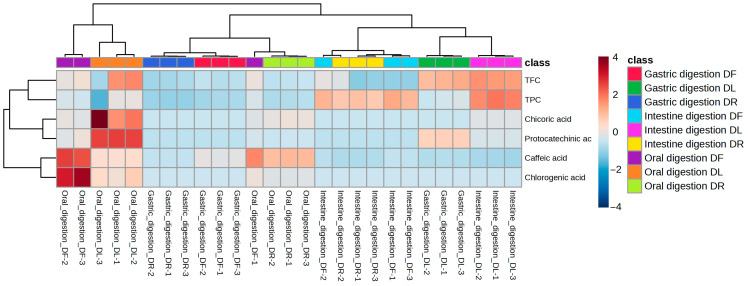
Heat map analysis of phenolic compound profiles in dandelion extracts across in vitro simulated digestion phases.

Generally, the concentration of phenolics in DL, both before and after digestion, was higher than that in DF and DR (Figure 3). This is consistent with the findings for total phenolic and flavonoid contents in DF, DR, and DL. However, the total amounts of monomeric phenolic compounds obtained from DF, DR, and DL at different stages of digestion were less than that at the undigested stage. This reduction may be attributed to the degradation or conversion of certain individual phenolic acids to other compounds during the digestion process, or the introduction of new compounds. For example, Laib et al. [49] observed a decrease in total polyphenols and tannins during gastric digestion of dandelion leaves, attributed to acid hydrolysis and matrix interactions. Among these, some unknown compounds may exist [45]; this corresponds with Corrêa et al.’s findings [13].

### 3.7. Neuroprotective Effects of Dandelion Polyphenols During Simulated Digestion

#### 3.7.1. AChE Inhibition Rate of Dandelion Polyphenols During Simulated Digestion

**Figure 4:**
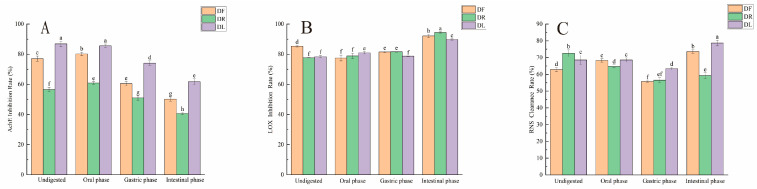
The inhibitory activity levels of dandelion extracts after simulated digestion in vitro: (**A**) acetylcholinesterase activity levels (AChE); (**B**) lipoxygenase activity levels (LOX); (**C**) RNS activity levels (RNS). Different letters indicate significant differences between groups (*p* < 0.05).

The rates of AChE inhibition by digestive fluids from different sites are shown in Figure 4A. After in vitro simulated digestion, the inhibition rate of AChE by all digestive fluids decreased significantly. The inhibition rates of AChE by DF digestate were in the following order: oral stage (80.15 ± 1.20%) > undigested (77.03 ± 1.76%) > gastric stage (60.44 ± 1.39%) > small intestine stage (50.19 ± 1.39%). (The IC_50_ values for DF, DR, DL, and the Huperzine A group were 0.428, 0.5326, 0.5532, and 0.3532, respectively.) A study of a freeze-dried extract of *Epilobium angustifolium* also showed that the gastric digestive phase had lower inhibitory activity against AChE than the small intestinal phase [50]. The inhibition by the digestive solution of DR was in the following order: oral stage (60.86 ± 0.90%) > undigested (56.54 ± 1.40%) > gastric stage (51.00 ± 1.55%) > small intestine stage (40.60 ± 0.72%). The inhibition by DL digestive fluid was in the following order: undigested (86.82 ± 1.74%) > oral phase (85.55 ± 1.18%) > gastric phase (74.10 ± 1.44%) > small intestinal phase (61.76 ± 1.87%). Zhang et al. [44] demonstrated that dandelion extracts retained partial antioxidant and anti-inflammatory activities after digestion, but their study did not involve neuroprotective targets such as AChE. A study on a freeze-dried extract of *Epilobium angustifolium* also showed that the gastric digestion phase inhibited AChE at a higher rate than the small intestine phase [50]. The decreased inhibition of AChE by the digestive fluids of DF, DR, and DL in the small intestinal stage may be because their antioxidant components (e.g., phenolic acids and flavonoids) are sensitive to alkaline environments. These components can undergo structural changes under alkaline conditions, thereby altering their chemical properties [51]. The increased inhibition of AChE by the digestive fluids of DF and DR in the oral phase compared to the undigested fluids may be attributed to the increase in the TPC and a decrease in the content of some phenolic compounds, including chlorogenic acid, which attenuates antagonistic interactions between polyphenolic compounds such as chlorogenic and caffeic acids [37]. In contrast, the digestive fluids of DL in the oral phase showed an increase in both TPC and TFC but the chlorogenic acid content did not change (Table 3). This is consistent with the fact that the inhibition rate of the digestive fluid in the oral phase was slightly lower than that of the undigested fluid.

#### 3.7.2. LOX Inhibition Rate of Dandelion Polyphenols During Simulated Digestion

Figure 4B shows that digestive fluids from different digestive sites significantly inhibited LOX. (The IC_50_ values for DF, DR, DL, and the Vc group were 0, 0.7893, 0, and 1.271, respectively.) DR had the highest LOX inhibition rate (94.50 ± 0.47%) in the small intestine stage, which significantly exceeded that of the undigested solution by 16.83% (*p* < 0.05). DL showed the highest LOX inhibition (80.90 ± 0.75%) at the oral stage, where the reduction in the protocatechuic acid content of the digestive fluid was statistically smaller compared to the other groups (Table 3). We hypothesised that the highest LOX inhibition was due to the enhanced inhibitory effect of protocatechuic acid at increasing concentrations [36]. The LOX inhibition rates of DR and DF in the gastric stage were similar, and both were higher than those of the digestive fluid of DL. Overall, the LOX inhibition rates in digestive fluids from various sites were higher than those in undigested fluids. Compounds such as protocatechuic acid, chlorogenic acid, and chicoric acid were not detected in the digestive fluid of DR at the intestinal stage using the UPLC-QTOF-MS/MS system, and only a small amount of caffeic acid was detected (Table 3). The content of certain phenolic compounds, such as with chlorogenic acid, exhibits a negative correlation with the pH of the digestive buffer during intestinal digestion [52]. The greater inhibition of LOX in digestive fluids during the intestinal digestion phase of DR may be related to the lower antagonistic effect of the active ingredient and the inhibitory effect of caffeic acid on LOX activity. Yu et al. found that caffeic acid inhibited 5-LOX at the optimum dosage (66 μM in vitro; 30 mg/kg in vivo), with no additional activity at higher levels [53]. Meanwhile, Durak et al. found that the antagonistic impact of LOX inhibition increased from an inhibition factor = 1.12 to 1.51 in an experiment imitating the influence of the gastrointestinal digestion environment on phenolic interaction in coconuts [54]. This is also consistent with the decreasing trend of LOX inhibition in digestive fluids from the oral and stomach phases of DF, which may be attributed to the antagonistic effect of the active chemicals that could enhance the efficiency of natural polyphenolase inhibitor development by optimising the digestive formulation.

#### 3.7.3. RNS Scavenging Rate of Dandelion Polyphenols During Simulated Digestion

As shown in Figure 4C, RNS clearance by digestive fluids from different digestive site sources varied significantly with the digestion stage. (The IC_50_ values for DF, DR, DL, and the Vc group were 0, 0.9755, 5.031, and 87.81, respectively.) The clearance of digestive fluids from DL and DF at the oral stage did not differ significantly, but both were significantly higher than that from DR at the oral stage, which was elevated by 2.56% (*p* < 0.05) compared with that in undigested samples. The lower digestive fluid clearance of DR, DL, and DF during the gastric phase may be attributed to the sensitivity of polyphenolic compounds to acidic conditions in the stomach [55]. This may also be related to the destruction of the phenolic structures by gastric acid. Thoma-Valdesa et al. found that gastric digestion reduced the number of compound species from 29 to 26 in native Chilean red strawberries [56]. The highest clearance of DL digestate (78.71 ± 1.66%) was observed in the small intestine stage, which was elevated by 11.42% (*p* < 0.05) compared to undigested conditions, indicating that digestion promotes polyphenol release, with the highest TPC (9398 ± 491.10 mg/100 g) increased by 135.8% compared to the undigested stage (Table 3). This may be due to a surge in polyphenol concentration which enhances inhibitory action on RNS. Prior research indicates that the RNS scavenging ability of *Citharexylum solanaceous* pulp + peel and seed extracts is directly and positively correlated with polyphenol concentration [57]. Furthermore, the surge in polyphenol release (+135.8%) from DL in the small intestine, where polyphenol activity was activated, resulted in the highest RNS clearance.

## 4. Conclusions

This study comprehensively evaluated the phenolic components and neuroprotective potential of different parts of dandelion (flower, root and leaf). The results showed that the contents of total phenols (39.87 mg/g) and total flavonoids (32.50 mg/g) in DL were the highest. A total of 84 polyphenols, including 38 phenolic acids and 39 flavonoids, were identified using UPLC-ESI-QTOF-MS. DF had the highest flavonoid content, whereas DL and DR had the highest phenolic acid content. Protocatechuic acid content was the highest in the flowers, roots, and leaves of dandelion, followed by chicory acid. In vitro neuroprotection experiments have shown that DL polyphenols have a strong inhibitory effect on acetylcholinesterase and lipoxygenase, whereas DF polyphenols have a strong RNS clearance ability. After simulated digestion in vitro, the TPC and TFC in DF, DR, and DL were significantly higher than before digestion. Polyphenols from different parts of dandelion exhibited the strongest inhibitory capacity against AChE. For LOX and RNS, polyphenols from dandelion showed the best inhibitory effect in the small intestine, followed by the oral and gastric stages. Additionally, at all digestive stages, the polyphenols in DL demonstrated significantly superior inhibitory effects on AChE compared to those in DF and DR. The above results revealed that dandelions have the potential to treat neurological diseases, with DL containing functional components that can prevent AD and oxidative stress-related diseases and be administered as a dietary intervention, suggesting that dandelion leaves are a promising functional food for such applications pending in vivo validation. Further research is warranted to assess the in vivo efficacy and bioavailability of dandelion polyphenols.

## Figures and Tables

**Table 1:** The contents of total polyphenols, flavonoids and monophenols in different parts of the dandelion.

	DF	DR	DL
TPC (mg GAE/100 g)	3354.00 ± 161.12 ^a^	1876.00 ± 341.29 ^b^	3986.67 ± 413.70 ^a^
TFC (mg RE/100 g)	2411.00 ± 249.94 ^b^	1512.00 ± 34.72 ^c^	3250.00 ± 118.04 ^a^
Protocatechuic acid (mg/100 g)	284.66 ± 63.48 ^c^	426.00 ± 63.18 ^b^	1899.88 ± 33.37 ^a^
Chlorogenic acid (mg/100 g)	1.00 ± 0.08 ^b^	2.11 ± 0.36 ^a^	0.12 ± 0.08 ^c^
Caffeic acid (mg/100 g)	2.04 ± 0.06 ^a^	1.28 ± 0.14 ^b^	0.99 ± 0.04 ^c^
Chicoric acid (mg/100 g)	35.66 ± 8.52 ^b^	62.88 ± 29.22 ^ab^	116.43 ± 35.89 ^a^
Rutin (mg/100 g)	17.00 ± 0.48 ^a^	16.90 ± 4.90 ^a^	3.00 ± 0.25 ^b^
Quercetin (mg/100 g)	0.15 ± 0.02 ^a^	2.01 ± 0.20 ^a^	0
Luteolin (mg/100 g)	8.37 ± 0.18 ^a^	0	0.11 ± 0.07 ^b^

Different letters indicate significant differences between different parts of the dandelion for the same compound (*p* < 0.05).

**Table 2:** Metabolites in UPLC-ESI-Q-TOF-MS dandelion extract.

Number	Accepted Description	Rt (min)	*m*/*z* (+)	Formula	Classification	Identification Confidence Level (ICL)
1	2-Hydroxy-4-methoxybenzaldehyde	1.992	108.02	C_8_H_8_O_3_	Aldehyde	Level 2a
2	4-Methylumbelliferyl *β*-*D*-glucuronide	4.436	353.09	C_16_H_16_O_9_	Coumarins	Level 2a
3	5,7-dihydroxycoumarin	0.740	179.03	C_9_H_6_O_4_	Coumarins	Level 1
4	6,8-Difluoro-7-hydroxy-4-methylcoumarin	1.580	423.05	C_10_H_6_F_2_O_3_	Coumarins	Level 2b
5	6-Hydroxycoumarin	8.276	117.03	C_9_H_6_O_3_	Coumarins	Level 1
6	Columbianetin acetate	4.757	577.20	C_17_H_16_O_5_	Coumarins	Level 2a
7	Isoimperatorin	6.337	309.21	C_16_H_14_O_4_	Coumarins	Level 1
8	12,13-Dihydroxy-9Z-octadecenoic acid	6.747	313.24	C_18_H_34_O_4_	Fatty acid	Level 2b
9	12a-Hydroxydalpanol	5.631	467.11	C_23_H_24_O_8_	Flavonoid	Level 2a
10	2′-Methoxyformonetin	1.801	321.07	C_17_H_14_O_5_	Flavonoid	Level 2a
11	3,5,7-Tetrahydroxy-6,8-dimethoxyflavone	5.996	691.13	C_17_H_14_O_8_	Flavonoid	Level 2b
12	3,5-Dimethoxy-3,5,7,4-tetrahydroxyflavone	4.423	345.06	C_17_H_14_O_8_	Flavonoid	Level 2b
13	3-Hydroxy-glabrol	1.184	409.00	C_25_H_28_O_5_	Flavonoid	Level 2a
14	4,5,7-Trihydroxy 3,3,6,8-tetramethoxyflavone	5.882	391.1	C_19_H_18_O_9_	Flavonoid	Level 2b
15	Nordalbergin	6.443	253.05	C_15_H_10_O_4_	Flavonoid	Level 2a
16	6-O-Methylscutellarin	2.226	625.14	C_27_H_30_O_17_	Flavonoid	Level 2a
17	Spinacetin 3-*O*-gentiobioside	2.289	693.16	C_29_H_34_O_18_	Flavonoid	Level 3
18	Haploside C	2.022	695.18	C_31_H_36_O_18_	Flavonoid	Level 3
19	(+)-Catechin	5.261	581.17	C_15_H_14_O_6_	Flavonoid	Level 1
20	Silychristin	6.194	433.09	C_25_H_22_O_10_	Flavonoid	Level 2a
21	Quercetin 3-*O*-malonylglucoside	3.901	1162.08	C_24_H_22_O_15_	Flavonoid	Level 2a
22	Quercetin 3-*α*-*L*-arabinopyranoside	2.091	473.03	C_20_H_18_O_10_	Flavonoid	Level 2a
23	Quercetin 3-*O*-arabinoside	3.867	433.07	C_20_H_18_O_11_	Flavonoid	Level 2a
24	Astragalin (Kaempferol 3-*O*-glucoside)	3.771	447.09	C_21_H_20_O_11_	Flavonoid	Level 1
25	3,8-Biapigenin	0.663	539.14	C_30_H_18_O_10_	Flavonoid	Level 2b
26	Tiliroside	2.482	633.10	C_30_H_26_O_13_	Flavonoid	Level 2a
27	Bavachinin	3.190	337.14	C_21_H_22_O_4_	Flavonoid	Level 2a
28	Chrysoeriol	5.972	299.06	C_16_H_12_O_6_	Flavonoid	Level 1
29	Maritimein	3.784	895.19	C_21_H_20_O_11_	Flavonoid	Level 2a
30	Luteolin	5.510	571.09	C_15_H_10_O_6_	Flavonoid	Level 1
31	Luteolin-7,3′-di-*O*-glucoside	3.194	609.14	C_27_H_30_O_16_	Flavonoid	Level 2a
32	Luteolin 7-*O*-glucoside	4.310	447.09	C_21_H_20_O_11_	Flavonoid	Level 1
34	Tricin	5.959	329.07	C_17_H_14_O_7_	Flavonoid	Level 1
35	Apigenin 7-*O*-glucuronide	5.890	269.04	C_21_H_18_O_11_	Flavonoid	Level 2a
36	Kaempferol 3-*O*-rhamnoside	3.642	593.14	C_27_H_30_O_15_	Flavonoid	Level 2a
37	Eriodictyol	0.681	289.07	C_15_H_12_O_6_	Flavonoid	Level 1
38	Rhamnetin	5.583	315.05	C_16_H_12_O_7_	Flavonoid	Level 1
39	Cirsimarin	5.659	477.14	C_23_H_24_O_11_	Flavonoid	Level 2a
40	Avicularin (Quercetin 4′-*O*-arabinoside)	2.090	473.03	C_20_H_18_O_11_	Flavonoid	Level 2a
41	Neodiosmin	4.586	631.167	C_28_H_32_O_15_	Flavonoid	Level 2a
42	Myricetin	4.093	635.06	C_15_H_10_O_8_	Flavonoid	Level 1
43	Isorhamnetin 3-*O*-neohesperidoside	3.900	659.13	C_24_H_22_O_15_	Flavonoid	Level 2a
44	Naringin dihydrochalcone	5.942	605.19	C_27_H_34_O_14_	Flavonoid	Level 2a
45	Tectoridin	4.572	497.08	C_22_H_22_O_11_	Flavonoid	Level 2a
46	Taxifolin	2.558	305.06	C_15_H_12_O_7_	Flavonoid	Level 1
47	Taxifolin 3-*O*-rhamnoside	5.119	468.15	C_21_H_22_O_11_	Flavonoid	Level 2a
48	5-O-Glucopyranosyloxy-3,4,7-trihydroxyneoflavone	3.771	483.07	C_21_H_20_O_11_	Flavonoid	Level 2b
49	Succinic acid	0.870	117.02	C_4_H_6_O_4_	Organic acid	Level 1
50	Maleic acid	0.754	115.00	C_4_H_4_O_4_	Organic acid	Level 1
51	Citric acid	0.721	191.02	C_6_H_8_O_7_	Organic acid	Level 1
52	1,3-Dicaffeoylquinic acid	2.067	353.09	C_25_H_24_O_12_	Phenolic acids	Level 2a
53	Cynarin (1,5-di-*O*-caffeoylquinic acid)	1.567	353.08	C_25_H_24_O_12_	Phenolic acids	Level 1
54	3,4-Di-*O*-galloylquinic acid	2.292	495.07	C_21_H_20_O_14_	Phenolic acids	Level 2a
55	3,4-Dihydroxyphenylacetic acid	2.240	334.07	C_8_H_8_O_4_	Phenolic acids	Level 1
56	3-Dehydroquinic acid	0.640	379.09	C_7_H_10_O_6_	Phenolic acids	Level 2a
57	3-*O*-*p*-Coumaroylquinic acid	2.640	337.09	C_16_H_18_O_8_	Phenolic acids	Level 2a
58	4-*O*-*trans*-caffeoylquinic acid	2.067	191.05	C_16_H_18_O_9_	Phenolic acids	Level 1
59	4-Hydroxy-3-methylbenzoic acid	5.941	151.04	C_8_H_8_O_3_	Phenolic acids	Level 1
60	4-Hydroxybenzoic acid	5.380	130.03	C_7_H_6_O_3_	Phenolic acids	Level 1
61	5,5′-(4-Tetradecene-1,4-diyl)bis[1,3-benzenediol]	6.926	435.25	C_26_H_36_O_4_	Phenolic acids	Level 2b
62	7-Hydroxycoumarin-4-acetic acid	4.745	441.08	C_11_H_8_O_5_	Phenolic acids	Level 2a
63	N1,N5,N10,N14-Tetra-trans-p-coumaroylspermine	5.938	785.35	C_46_H_50_N_4_O_8_	Phenolic acids	Level 2b
64	3-Hydroxyphysodic acid	6.080	115.00	C_24_H_26_O_10_	Phenolic acids	Level 2b
65	Confluentic acid	5.944	523.23	C_28_H_36_O_8_	Phenolic acids	Level 2b
66	Chicoric acid	3.608	473.07	C_22_H_18_O_12_	Phenolic acids	Level 1
67	Caftaric acid	4.615	149.01	C_13_H_12_O_9_	Phenolic acids	Level 1
68	Ethyl trans-caffeate	5.810	207.07	C_11_H_12_O_4_	Phenolic acids	Level 1
69	Chlorogenic acid (5-*O*-caffeoylquinic acid)	1.545	353.09	C_16_H_18_O_9_	Phenolic acids	Level 1
70	Gallic acid	7.217	439.27	C_7_H_6_O_5_	Phenolic acids	Level 1
71	Matairesinoside	2.618	555.16	C_26_H_32_O_11_	Phenolic acids	Level 2a
72	trans-Cinnamic acid	0.606	131.05	C_9_H_8_O_2_	Phenolic acids	Level 1
73	Daphnin	1.764	339.07	C_15_H_16_O_9_	Phenolic acids	Level 2a
74	Chlorogenic acid isomer (3-*O*-caffeoylquinic acid)	4.092	353.09	C_16_H_18_O_9_	Phenolic acids	Level
75	Bergenin	1.219	329.09	C_14_H_16_O_9_	Phenolic acids	Level 1
76	3,5-Dicaffeoylquinic acid	4.092	515.12	C_25_H_24_O_12_	Phenolic acids	Level 2a
77	Isochlorogenic acid B (3,4-dicaffeoylquinic acid)	4.423	515.12	C_25_H_24_O_12_	Phenolic acids	Level 2a
78	Cryptochlorogenic acid (4-*O*-caffeoylquinic acid)	2.471	179.03	C_16_H_18_O_9_	Phenolic acids	Level 1
79	3,4-Dihydroxybenzaldehyde	1.992	137.02	C_7_H_6_O_3_	Phenolic acids	Level 1
80	Verminoside	4.886	523.15	C_24_H_28_O_13_	Phenolic acids	Level 2a
81	(+)-Abscisic acid (cis-isomer)	5.194	348.10	C_15_H_20_O_4_	Phenolic acids	Level 1
82	4,5-Di-O-caffeoylquinic acid methyl ester	4.832	531.15	C_26_H_26_O_12_	Phenolic acids	Level 2a
83	Pentagalloylglucose	8.136	769.09	C_41_H_32_O_26_	Tannins	Level 2b
84	(+)-Abscisic acid (trans-isomer)	5.194	348.10	C_15_H_20_O_4_	Terpenoid	Level 1

**Table 3:** In vitro simulated total phenol, flavonoid and monophenol contents in different parts of dandelion after digestion.

	TPC (mg GAE/100 g)	TFC (mg RE/100 g)	Protocatechinic Acid (mg/100 g)	Chlorogenic Acid (mg/100 g)	Caffeic Acid (mg/100 g)	Chicoric Acid (mg/100 g)
Oral digestion DF	3902.00 ± 254.98 ^b^	1514.00 ± 122.60 ^b^	133.20 ± 51.94 ^b^	0.40 ± 0.29 ^a^	0.48 ± 0.06 ^a^	7.19 ± 1.11 ^b^
Oral digestion DR	2824.67 ± 140.50 ^c^	793.00 ± 60.53 ^c^	43.13 ± 5.16 ^c^	0.04 ± 0.00 ^b^	0.31 ± 0.01 ^b^	9.90 ± 1.25 ^b^
Oral digestion DL	4476.00 ± 215.72 ^a^	3601.33 ± 102.24 ^a^	932.00 ± 3.74 ^a^	0.12 ± 0.04 ^ab^	0.21 ± 0.01 ^c^	61.99 ± 27.08 ^a^
Gastric digestion DF	2467.00 ± 131.04 ^b^	698.00 ± 75.01 ^b^	0	0	0.15 ± 0.00 ^a^	0
Gastric digestion DR	2072.00 ± 105.06 ^c^	380.00 ± 79.62 ^c^	0	0	0.10 ± 0.00 ^b^	0
Gastric digestion DL	3796.67 ± 280.08 ^a^	2885.67 ± 183.51 ^a^	350.48 ± 3.48 ^a^	0	0.07 ± 0.00 ^c^	0
Intestine digestion DF	7620.00 ± 399.25 ^b^	1541.00 ± 74.47 ^b^	0	0	0.09 ± 0.01 ^a^	0.38 ± 0.00 ^b^
Intestine digestion DR	6726.00 ± 202.16 ^c^	895.00 ± 98.22 ^c^	0	0	0.06 ± 0.01 ^b^	0
Intestine digestion DL	9398.00 ± 491.10 ^a^	3245.67 ± 125.17 ^a^	95.58 ± 0.28 ^a^	0	0.05 ± 0.00 ^c^	6.63 ± 3.23 ^a^

Different letters indicate significant differences between different parts of the dandelion for the same compound after in vitro simulated digestion (*p* < 0.05).

## Data Availability

The original contributions presented in this study are included in the article. Further inquiries can be directed to the corresponding author.
